# Eosinophil granuloma due to parasite treated by laparoscopic and endoscopic cooperative surgery: a case report

**DOI:** 10.1186/s40792-020-0772-9

**Published:** 2020-01-10

**Authors:** Toshikatsu Tsuji, Noriyuki Inaki

**Affiliations:** 1 Department of Gastroenterological Surgery, Ishikawa Prefectural Central Hospital, 2-1 Kuratsukihigashi, Kanazawa, Ishikawa 9208530 Japan; 2 Department of Surgery, Juntendo Urayasu Hospital, Juntendo University, 2-1-1, Tomioka, Urayasu-shi, Chiba 2790021 Japan

**Keywords:** Gastric eosinophilic granuloma, Parasitic infection, Anisakiasis, Laparoscopic and endoscopic cooperative surgery (LECS)

## Abstract

**Background:**

Gastric eosinophilic granuloma caused by parasitic infection is rare. It is often suspected to be a malignant disease and it is difficult to diagnose. We successfully diagnosed and removed a gastric eosinophilic granuloma using laparoscopic and endoscopic cooperative surgery (LECS).

**Case presentation:**

A 35-year-old woman visited our hospital because of epigastric pain. Upper gastrointestinal endoscopy revealed a 15 mm submucosal tumor (SMT) with changes in the folds, such as enlargement and convergence, located in the greater curvature of the lower gastric body. Computed tomography (CT) showed a dense, nonenhanced area of 15 mm at the same site. SMT was suspected, but undifferentiated cancer could not be excluded. We performed laparoscopic partial gastrectomy using LECS for resection biopsy.

Histopathological examination showed an SMT 8 × 8 × 5 mm in size with an unclear boundary and necrosed insects at the core of the tumor. There was marked eosinophilic infiltration around the area. The diagnosis was gastric granuloma caused by parasitic infection.

**Conclusions:**

It is difficult to differentiate gastric eosinophilic granuloma caused by parasitic infection from malignant disease. In this case, LECS is considered a minimally invasive and useful procedure.

## Background

Anisakiasis is a parasitic disease caused by the consumption of raw fish infected with *Anisakis*. This disease has been reported in Japan, Korea, the Netherlands, and western Europe, where raw fish is eaten. Gastric anisakiasis is divided into two types: the acute type (acute anisakiasis) and the chronic type (eosinophilic granuloma). The acute type, which develops sudden severe abdominal pain, is well known. The chronic type is relatively rare, accounting for 2~4% of anisakiasis cases. It is difficult to differentiate the chronic type of anisakiasis from malignant disease by endoscopy and imaging to achieve a definitive diagnosis before surgery. We performed laparoscopic and endoscopic cooperative surgery (LECS) for therapeutic diagnosis, which is considered a minimally invasive and useful procedure in this disease.

## Case presentation

**Fig. 1:**
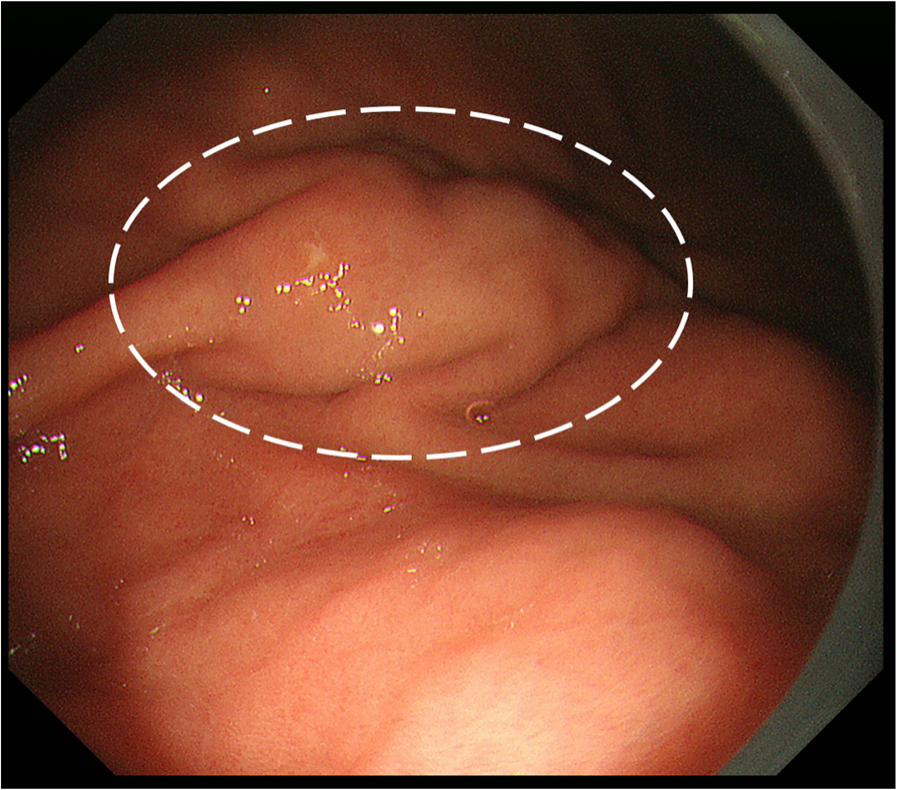
Upper gastrointestinal endoscopy findings: 15 mm SMT with the changes in the fold such as enlargement and convergence located in the greater curvature of the lower gastric body. The mucosa was partially erosive

**Fig. 2:**
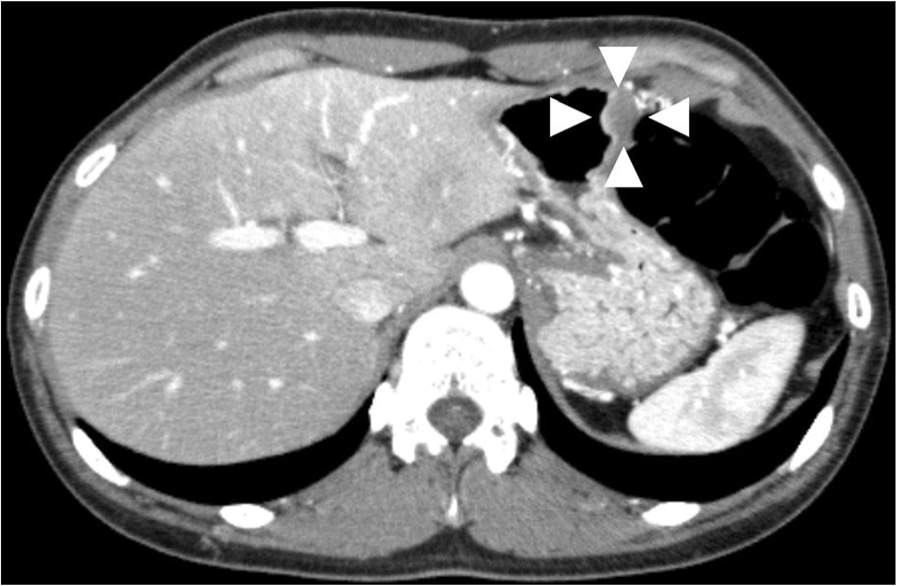
Enhanced abdominal CT findings. The dense stain defective area of 15 mm at the greater curvature of the gastric body

A 35-year-old woman visited our hospital because of epigastric pain. Laboratory data showed white blood cell count of 5.83 × 10^3^/μL, eosinophils 3.8%. Upper gastrointestinal endoscopy revealed a 15 mm SMT with changes in the folds, such as enlargement and convergence, located in the greater curvature of the lower gastric body (Fig. 1). The mucosa was partially erosive, and the tumor was elastic and hard. Endoscopic ultrasound (EUS) findings revealed no regions that could be identified as a tumor at either 12 MHz or 20 MHz, and the layered structure was maintained. CT showed a localized nonenhanced nodule 15 mm in size at the same site (Fig. 2). Thus, gastric SMT was suspected, but undifferentiated cancer could not be excluded. Biopsy was performed several times, and deep biopsy was also performed, but no malignant findings were observed. We planned to carry out total biopsy after obtaining informed consent because malignant disease could not be excluded.

**Fig. 3:**
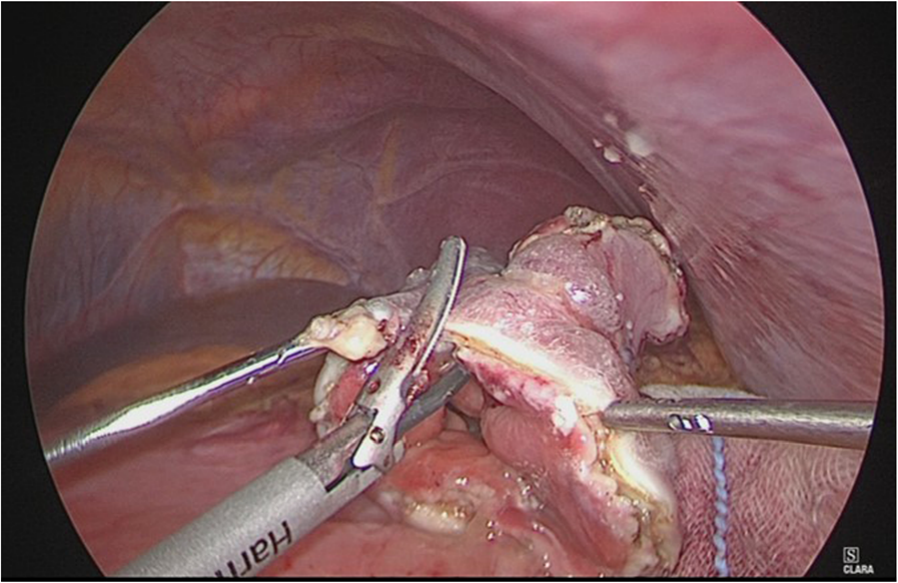
Operative findings. After mucosal incision, the specimen was circumferentially resected by using laparoscopic coagulating shears

We performed laparoscopic partial gastrectomy via LECS. The port arrangement was shifted to the foot side relative to the port arrangement in conventional gastrectomy because of severe gastroptosis. Five ports (one 12-mm, one 5-mm, and three 2-mm ports) were placed. The tumor location was confirmed in the greater curvature of the lower gastric body by endoscopy. The omentum around the tumor was minimally removed. A mucosal incision was made around the tumor with an adequate margin. The most anal side of the mucosal incision was endoscopically perforated. The rest of the specimen was circumferentially resected using laparoscopic coagulating shears (Fig. 3). The specimen was stored in a plastic bag and orally extracted. The defect in the stomach wall was closed with continuous full-layer suturing using barbed sutures (3-0 V-LOC^TM^ 180, Medtronic plc, Dublin, Ireland).

The patient was discharged on the seventh postoperative day without complications. At the 6-month postoperative checkup, she had no complaints, such as bloating, pyrosis, and burp.

**Fig. 4:**
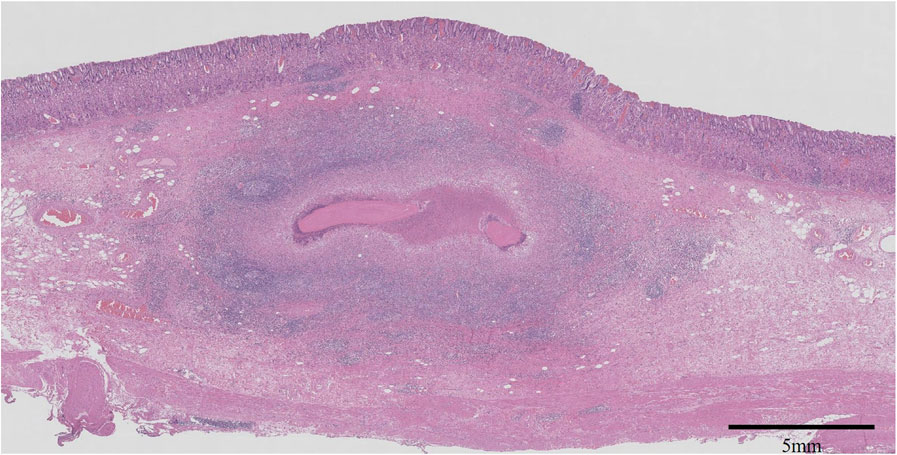
Histopathological findings. 8 × 8 × 5 mm submucosal tumor with unclear boundary was observed and necrosed insect was found as the core of the tumor. There was marked eosinophil infiltration around the area

Histopathological examination showed a yellowish white SMT 8 × 8 × 5 mm in size with an unclear boundary, and necrosed insects were found at the core of the tumor. There was marked eosinophilic infiltration around the area. The final diagnosis was gastric granuloma caused by parasitic infection (Fig. 4).

## Discussion

In our case, therapeutic diagnosis was less invasively performed by LECS for the granulomatous tumor which is difficult to differentiate as malignant tumor. The histopathological findings revealed that eosinophilic granuloma formation due to a chronic foreign body reaction. In this kind of chronic type, an EUS usually reveals a central hyperechoic area, which is a characteristic finding of Anisakis granuloma [1]. It also represents a circular or elliptical structure with hyperechoic signals in the center of a hypoechoic mass with an unclear boundary.

However, these typical findings could not be confirmed in our case. Its endoscopic finding revealed a somewhat malignant tumor (e.g., gastrointestinal stromal tumor (GIST), metastatic gastric tumor) with ulcer formation and changes in the folds, such as enlargement, convergence, and erosion, on its top [2]. Thus, we ultimately suspected malignancy; biopsy was performed several times, which resulted in failure to diagnose. Although we considered gastrectomy with lymphadenectomy in case of malignant tumor, we finally decided to perform LECS for the therapeutic total biopsy. As a result, we could obtain a definitive diagnosis without complications and patient could be treated less invasively preserving almost the whole stomach.

LECS is a surgical technique for SMT removal reported by Hiki et al. and a procedure that can minimize deformation and dysfunction by avoiding excessive resection of the stomach wall. However, when obvious ulcers are involved, it is necessary to prevent the spread of the tumor cells, and it may be better to consider the following methods that do not expose the tumor to the abdominal cavity: inverted LECS, nonexposed endoscopic wall inversion surgery (NEWS), and a combination of laparoscopic and endoscopic approaches to neoplasia with a nonexposure technique (CLEAN-NET) [3–7].

We chose original LECS procedure and perforated gastric wall during resection of the tumor; however, we carefully paid attention not to spoil the gastric juice out from the stomach and to let the surface of the tumor touch to the abdominal cavity. The diagnosis was gastric granuloma caused by parasitic infection, excluding malignancy or typical SMT such as GIST and leiomyoma. LECS was a valid procedure for this case as a result.

The patient did not eat raw fish 1 week before she visited at least. However, she usually eats raw fish. It is also important to keep in mind the gastric eosinophilic granuloma caused by parasitic infection, especially in regions where raw fish is eaten.

## Conclusions

LECS is a favorable less invasive candidate procedure for therapeutic diagnosis when we encounter the granuloma which is difficult to diagnose.

## Data Availability

Data sharing is not applicable to this article, because no datasets were generated or analyzed during this study.

## Abbreviations

CLEAN-NET: Combination of laparoscopic and endoscopic approaches to neoplasia with a nonexposure technique
CT: Computed tomography
EUS: Enhanced ultrasound
GIST: Gastrointestinal stromal tumor
LECS: Laparoscopic and endoscopic cooperative surgery
NEWS: Nonexposed endoscopic wall inversion surgery
SMT: Submucosal tumor
